# Surgical management of melanoma: an EORTC Melanoma Group survey

**DOI:** 10.3332/ecancer.2013.294

**Published:** 2013-03-28

**Authors:** A Testori, J Soteldo, B Powell, F Sales, L Borgognoni, P Rutkowski, F Lejeune, PAM van Leeuwen, A Eggermont

**Affiliations:** 1 European Institute of Oncology, Italy; 2 Hospital De Clinicas, Caracas, Venezuela; 3 St. Georges Hospital, UK; 4 Institut Jules Bordet, Brussels; 5 Centro Regionale di Riferimento per il Melanoma, Ospedale S. M. Annunziata, Italy; 6 Centrum Onkologii Instytut im Marii, Sklodowskiej-Curie Warsawa, Poland; 7 Centre Hospitalier Universitaire Vaudois, Switzerland; 8 University Amsterdam, The Netherlands; 9 Institute Gustave Roussy Paris, France

## Abstract

**Objectives:**

The objective of the article is to explore the surgical practices and views in the treatment of melanoma within members and non-members of the EORTC Melanoma Group (MG) during the years 2003–2005.

**Methods:**

An e-mail questionnaire (see appendix) developed within the EORTC MG was sent to all melanoma units (MUs) of the EORTC (180) and to selected international centres between 2003 and 2005. The questionnaire investigated the different practices regarding surgical management of melanoma patients at all stages.

**Results:**

A total of 75 questionnaires were returned from centres in Europe (70), Israel (3), Australia (1) and the United States (1). Resection margins on primary melanoma vary according to AJCC 2002 staging. Sixty three of 75 MUs perform Sentinel node biopsy. Modified radical neck dissection is performed in 82% of MUs for macrometastases and in 80% of MUs for micrometastases. Most MUs surveyed perform all three levels of Berg axillary dissection whether for macrometastases (79%) or micrometastases (62%). An ilio inguinal-obturator dissection is proposed with macrometastases (41% of MUs), whereas 33% of MUs perform a pelvic dissection only if the Cloquet node is positive. Twenty five of 75 MUs perform an isolated limb perfusion with a therapeutic indication; three also as an adjuvant. The majority of MUs perform surgery for distant metastases including superficial (53 of 75 [71%]) or solitary visceral metastases (52 of 75[69%]) or for palliation (58 of 75[77%]).

**Conclusion:**

The adequacy of surgery appears to be the most important milestone in the therapeutic approach of melanoma. Even if surgery is fundamental in the different stages of the disease, there is quite a variability concerning the extension of the surgical treatment related to primary and lymphnodal disease. Phase III randomised trials have shown that wide margins, elective lymph node dissections, and prophylactic isolated limb perfusions have not improved survival and cannot be considered the standard of care in the routine management of primary melanoma. The surgical subgroup of the EORTC Melanoma Group is developing a new version of the surgical survey questionnaire including new treatment modalities like isolated limb infusion and electrochemotherapy, which were not frequently in use some years ago, to obtain new data to be compared to the nearly ten-year-old data.

## Participating physicians:

Omgo E Nieweg, MD (The Netherlands Cancer Institute); Prof. Dummer, MD (Dept.of Dermatology, University Hospital of Zurich); P Rutkowski, MD (Cancer Center Warsw, Poland); Prof. Bernhard Zelger, MD (Department of Dermatology, University of Innsbruck); G Mamelle, MD (Instituto Gustune Roussy, france); F Egli, MD (Surgical Department Kantonsspital 7000 Chur); Marco Hocevar, MD. PhD (Institute of Oncology, Jublsana, Slovenia); Prof. H Gollnick , MD; Prof. Hoekstra, MD ( EORTC 419, Magdeburg); Verschaeve Vincent , MD (Clinique Notre Dame de Grace); Prof. W Tilgen, MD (The Saarlandes University Hospital); Trotter J, MD (Royal Perth Hospital-Department of medical oncology, Australia); Prof. R Vanwijck, MD (Groupe Melanome Cliniques Universitaires st. Luc. Brussels); Prof. Sidika Kurul, MD (Istambul University, Institute of oncology.); L Kretschmer, MD (Department of dematology, University of Gottingen); Lorenzo Borgognoni, MD (Centro Regionale di Riferimento per il Melanoma. Ospedale S. M. Annunziata, Italy); Haim Gutman, M.D (Rabin Medical Center, Beilinson Campus); F Piette, MD (Lille (FRANCE) EORTC 254); César Martins, MD (Hospital de Santarém, Portugal); Thorsten Wegner, MD (Dermatologie-Charité-Universitätsklinikum der Humboldt-Universität Berlin); Bonenkamp, MD (UMC St. Radboud, Nijmegen. Netherlands); R Mackie, MD (University of Glasgow); Marsden J, MD (University Hospital Birmingham-Selley Oak Hospital); Alessandro Testori, MD (European Institute of Oncology, Italy); Prof. Marcello Pace, MD (University of Florence); Prof. Karsten Neuber, MD (Department of dermatology, University Hospital Eppendorf, Hamburg); F Truchetet - Metz Thionville, MD (EORTC Center 424); Prof. Dirk Schadendorf, MD (University Hospital Mannheim; Skin Cancer Unit); Marguerite Stas, MD (Surgical Oncology Gasthuisberg, Belgium); T Meyer, MD (Klinikum der Universität Erlangen-Nürnberg); Pippa Corrie, MD (UK, Cambridge EORTC 632); Prof. Radan Dzodic, MD (nstitut Za Onkologiju I Radiologiju Rbije, Servia); Borel-Rinkes, MD (Universitair Medisch Centrum-Academisch Ziekenhuis); Poul Harboe M.D (Odense University Hospital, Denmark ); Pavlotsky Felix, MD (Chaim Sheba Medical Center, Israel); Istvan Juhasz MD PhD (Dept of Dermatology University Debrecen, Hungary); Kristoffer T Drzewiecki, MD (University Hospital Rigshospitalet Copenhagen, Denmark); Prof. P Van Leeuwen, MD (Vrije U University Medical Center, Amsterdam); Raquel Andres, MD (Hospital Clinico Universitario Lozano Blesa, Spain); (Christiane Voit, MD Humbolt University of Berelin, EORTC 198 ); Prof. ES. Klein, MD (Sapir Medical Center Tel-Aviv University Israel); Meirion Thomas, MD (Royal Marsden Hospital, UK); Sales F, MD (Institut Jules Bordet, Belgium); Prof. Ferdy J Lejeune, MD (Centre Hospitalier Universitaire Vaudois (CHUV), Lausanne, Switzerland); JL. Perrot, MD (C.H.U. De Saint-Etienne-Hospital Nord, France); Perks A, MD (Nottimgham City hospital); Alexander Eggermont, MD PhD ( Erasmus University Medical center, Rotterdam);Chao D, MD (Royal Free Hospital); Saiag F Pr, MD (C.H.U. Ambroise Pare); Davies M, MD (Aberdeen, UK), Prof. Mozzillo N, MD (Istituto dei Tumori di Napoli G. Pascale, Italy); Prof. Janos Hunyadi, MD PhD Dsci (University Medical School, Dept. of dermatology, Debrecen, Hungary); Pablo Ortiz Romero, MD(Hospital 12 de Octubre, Spain); Donald L Morton, M.D PhD (John Wayne Cancer Institute, USA); Garcia Bartels, MD (Charite Campus Mitte, Germany); Th Horn, MD (Klinikum Krefeld, Germany); Giuseppe Giudice, MD (Università degli Studi di Bari, Italy); Mr NK James (Lister Hospital,Coreys Mill Lane, Stevenage, UK); Koller J MD (Landeskliniken Salzburg – St. Johanns-Spital, Austria); Prof. Kerl Begged, MD (Department of Dermatology Medical University of Graz, Austria).

## Introduction 

Surgery is the only effective treatment for melanoma patients. More than 95% of cures are achieved by surgery, and, therefore, a high standard is fundamental to guarantee the best clinical outcome.

Early diagnosis of melanoma is becoming more frequent, and, accordingly, minimally invasive yet oncologically sound surgery has become a priority in order to reduce morbidity and improve long-term quality of life. As a result, surgery for localised melanoma has steadily become less radical over the last three decades, and wide resection margins, elective lymph node dissection and prophylactic limb perfusion have largely been abandoned as a result of randomised phase III trials [1–6]. However, there remain international differences in guidelines for the surgical management of melanoma. The EORTC Melanoma Group (MG) has prepared a survey for melanoma units (MUs) collaborating as either active members (AMs) or probationary members (PMs), or not being directly involved within the MG studies. The aim of this survey has been to explore the current surgical practices and views of AMs and PMs of the EORTC MG and foreign melanoma units (FMUs). 

## Materials and methods

An e-mail questionnaire (see appendix) developed within the EORTC was sent to all MUs of the EORTC (175 MUs) and to selected international centres (five) between the Melanoma Spring Meetings of 2003 and 2005. The response rate was 42% (75 MUs). All questions were of the check-box type. The questionnaire investigated the different practices regarding resection margins, sentinel node biopsy (SNB), lymph node dissection (neck, groin and axillary), surgical management of distant metastases and the use of isolated limb perfusion in melanoma patients. Chi-squared tests were used to investigate differences among EORTC types of MUs. 

## Results

A total of 75 questionnaires were returned from centres in Europe (70), Israel (3), Australia (1) and the United States (1). We had 21 EORTC AMs, 35 PMs, and 19 FMUs. A further five centres returning questionnaires could not be identified and were excluded from the analyses.

### Primary melanoma resection margins

Answers for resection margins were given indicating a precise number of centimetres or by intervals (Table 1). The range of resection margins for *in situ *lesions varied from 0.2 to 1 cm with 71% of MUs performing a 0.5 cm margin (86% of AMs, 71% of PMs and 68% of FMUs), 96% of MUs from 0.5 to 1 cm. The range of resection margins for T1 lesions was 0.5–2 cm with 88% of MUs performing a 1-cm margin (90% of AMs, 94% of PMs, and 73% of FMUs), 96% of MUs from 0.5 to 1 cm. For T2 lesions, the range was 1–3 cm with 53% of MUs performing a 2-cm margin (57% of the AMs, 46% of PMs, and 64% of FMUs), 33% of MUs performing a 1–1.5-cm margin (38% of AMs, 34% of PMs, and 26% of FMUs), 97% of MUs from 1 to 2 cm. For T3 melanoma, the range was 1–4 cm with 71% of MUs performing a 2-cm margin (with significant differences between groups: 76% of AMs, 83% of PMs and 53% of FMUs, P = 0.05), 88% of MUs from 2 to 3 cm. For T4 lesions, the range was 1–4 cm with 49% of MUs performing a 2-cm margin (with highly significant differences between groups: 52% of AMs, 15% of PMs and 42% of FMUs, P = 0.007), 90% of MUs from 2 to 4 cm (38% of the AMs, 40% of PMs, and 53% of FMUs). 

### Sentinel lymph node biopsy (SNB)

**Figure 1:**
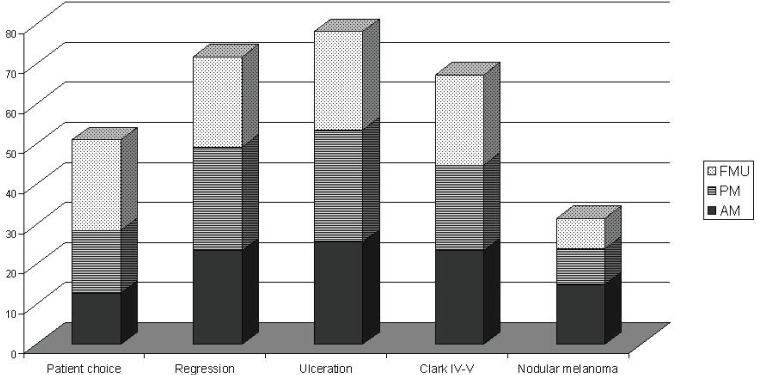
SNB for Breslow<1mm according to the EORTC status

Eighty four per cent of MUs perform SNB (81% of AMs, 88% of PMs, and 79% FMUs). The most common method used to perform SNB was Patent blue dye and Gamma Probe in combination (81% of MUs) (88% of the AMs, 77% of the PMs, and 80% of FMUs). The remaining members only used either Patent blue dye (3% of MUs: 6% of the EORTC AMs and 7% of FMUs), or the Gamma Probe (16% of MUs: 6% of the AMs, 23% of the PMs, and 13% of FMUs). Among the MUs performing SNB, the procedure is performed for all tumours between 1 and 4 mm thick. Fourteen per cent of MUs also perform SNB for lesions thinner than 1 mm (12% of the AMs, 26% of the PMs, and 27% of FMUs) and 90% of MUs also perform it for lesions thicker than 4 mm (17% of the AMs, 27% of the PMs, and 13% of FMUs). Fifty four MUs perform SNB for lesions thinner than 1 mm if one or more of the following factors are present: patient choice (with significant differences among groups: 29% of AMs, 40% of PMs, and 63% of FMUs; P = 0.03), regression (52% of AMs, 66% of PMs, and 63% of FMUs), ulceration (57% of AMs, 71% of PMs, and 68% FMUs), Clark IV–V lesions (52% of AMs, 54% of PMs, and 63% FMUs), and nodular melanoma (33% of AMs, 23% of PMs, and 21% FMUs) (Table 2). In Figure 1, we show percentages, referring to the overall choices of the EORTC MU groups; from the histogram we can see that the main reason for SNB was ulceration, and nodular melanoma was less frequent. Except for patient choice, which was a more frequent reason considered by FMUs, no significant differences among groups were observed. 

### Surgical approach regarding neck, axillary and inguinal and pelvic dissection

The analysis of therapeutic lymph node dissection (TLND) for positive nodes was considered only for MUs performing SNB (Table 3). 

#### Neck

Eighty two per cent of MUs (76% of the AM, 80% of the PM and 86% of FMUs) perform a modified radical neck dissection (MRND) for macrometastases following SNB. This procedure includes a cervical lymphadenectomy of levels I to V, sparing the accessory spinal nerve, internal jugular vein, and sternocleidomastoid. For micrometastases, 81% MUs (76% of the AMs, 77% of the PMs, and 86% of FMUs) perform an MRND, whereas 16% of MUs (20% of the AMs, 16% of the PMs, and 13% of FMUs) perform a limited neck dissection (LND), characterised by dissection of the nodal levels adjacent to the metastasis. When the SNB is only positive on immunohistochemistry (IHC), 55% of MUs (with significant difference among groups: 23% of the AMs, 58% of the PMs, and 60% of FMUs; P = 0.05) perform an MRND and 19% of MUs (29% of the AMs, 16% of the PMs, and 13% of FMUs) perform an LND. Only 6% of MUs (three EORTC PMs and one foreign) perform a TLND for PCR positive SNB (two PMs perform an LND, and another PM and one foreign unit perform an MRND).

Fifty one per cent of MUs perform a superficial parotidectomy for documented infraparotid metastases (19% of the AMs, 42% of the PMs, and 47% of FMUs), whereas 13% of MUs (10% of the AMs, 14% of the PMs, and 5% of FMUs) would also perform the procedure for a primary tumour located between the zygoma and the mastoid. 

#### Axilla

Most MUs surveyed perform all three levels of Berg axillary dissection whether for macrometastases 79% (94% of the AMs, 70% of the PMs, and 80% of FMUs) or micrometastases 62% (76% of the AMs, 51% of the PMs, and 66% of FMUs) on SNB. If the SNB is positive only on IHC, 48% of MUs (47% of the AMs, 48% of the PMs, and 46% of FMUs) perform a level-three dissection, whereas 24% (12% of the AMs, 29% of the PMs, and 27% of FMUs) perform a level-two dissection. For PCR positive sentinel nodes, only 8% of MUs perform a TLND. Three MUs (two PMs and one foreign member) perform a level-two dissection, and two MUs (two PMs) perform a level-three dissection. 

#### Groin

The ilioinguinal dissection (IID) is the most specialistic surgical lymph node dissection in melanoma patients. It is performed in 65% of MUs (with significant differences among groups: 82% of the AMs, 48% of the PMs, and 79% of FMUs; P = 0.02) when the SNB is positive for macrometastases, although 33% of MUs (with significant differences among groups: 17% of the AMs, 48% of the PMs, and 20% of FMUs, P = 0.05) perform a superficial inguinal dissection with a deep inguinal dissection being performed only when the Cloquet’s node is positive. When the SNB is positive for micrometastases, 60% of MUs (64% of the AMs, 55% of the PMs, and 59% of FMUs) perform an IID, whereas 25% of MUs (12% of the AMs, 32% of the PMs, and 26% of FMUs) perform a superficial inguinal dissection with a deep inguinal dissection being performed only when the Cloquet’s node is positive. If the SNB is positive on IHC, 8% of MUs (0% of the AMs, 10% of the PMs, and 13% of FMUs) perform an IID and 21% of MUs (0% of the AMs, 35% of the PMs, and 30% of FMUs) perform a superficial inguinal dissection with a deep inguinal dissection being performed only when the Cloque’s node is positive. For PCR positive nodes, only two MUs (EORTC one AM, one probationary) perform a superficial inguinal dissection, and three MUs (EORTC one AM, one PM, one FMU) perform an IID. Just one MU performs a superficial inguinal dissection with a deep inguinal dissection being performed only when the Cloquet’s node is positive. 

### Isolated limb perfusion (ILP)

Thirty three per cent MUs surveyed perform isolated limb perfusion (with significant differences among groups: 66% of the AMs, 20% of the PMs, and 36% of FMUs; P = 0.006) and the most common indication for ILP was therapeutic (100% of MUs). Only three MUs (12%) also perform this procedure as an adjuvant to surgery (three EORTC AMs).

Of the 50 MUs not performing ILP, 4% of PMs refer all patients with in-transit metastases to a centre performing ILP, whereas 72% of MUs (100% of EORTC AMs, 90% of PMs, and 100% foreign) only refer patients with inoperable or rapidly recurring in-transit metastasis. Twenty four per cent of MUs not performing ILP (10% of EORTC AMs, 20% of PMs, and 16% foreign) do not refer patients as these units prefer surgery, systemic therapy (5% active and 6% PMs) or both (67%) (5% of EORTC AMs, 11% PMs, 16% foreign). 

### Surgery for distant metastases

The majority of MUs perform surgery for distant metastases including superficial (with significant differences among groups: 52% of the AMs, 66% of the PMs, and 100% of FMUs; P = 0.01) or solitary visceral metastases (62% of the AMs, 80% of the PMs, and 57% of FMUs) or for palliation (81% of the AMs, 85% of the PMs, and 57% of FMUs). Seventeen MUs operate on patients with multiple visceral metastases (33% of the AMs, 17% of the PMs, and 21% of FMUs), either as required in clinical trials (17 MUs: 19% of the AMs, 25% of the PMs, and 21% of FMUs) or for surgical emergencies (22 MUs: 29% of the AMs, 34% of the PMs, and 21% of FMUs).

## Discussion

### Adjuvant surgical procedures for primary melanoma 

#### Margins

The resection margins for primary melanoma still differ between centres. The major differences are for T2 tumours where one-third of centres perform a 1-cm margin and two-thirds MUs perform a 2-cm margin. For thinner or thicker lesions, the proposed margins are 1 and 2 cm, respectively.

A 5-cm excision margin was challenged following the historical paper by Handley and supported by Breslow’s study that showed prognosis was related to the thickness of the primary lesion. Many randomised trials regarding margins have been conducted and are summarised in Table 4. Three trials included patients with melanomas less than 2 mm thick. In these trials, patients were randomised to an excision margin of 1 or 3 cm in the WHO-10 Trial [1, 2], and a margin of 2 cm was compared to 5 cm in separate French [3] and Scandinavian trials [4]. The Intergroup Trial in the United States [5, 6] randomised patients with melanomas 1–4 mm thick to either a 2 or 4 cm excision margin. These trials consistently showed that local recurrence rates, disease-free survival (DFS) and overall survival (OS) were virtually identical in the narrow and wide excision arms. However, these trials did not address excision margins of 1 and 2 cm in an attempt to determine a universal margin for all primary melanomas. 

A large non-randomised study similarly suggested that a 2-cm margin was adequate for melanomas thicker than 4 mm [7]. Thus a 1-cm margin appears to be adequate for melanomas less than 2 mm and a 2-cm margin for thicker melanomas. Consequently, split skin grafts are rarely necessary. Controversy was caused by the results of the UK-Melanoma Study Group trial [8], which compared excision margins of 1 cm with 3 cm for melanomas thicker than 2 mm. Nine hundred patients were randomised in this study. This trial showed that again the outcome was equal regardless of the margin. There was significantly more locoregional disease (local recurrences, and in-transit and regional lymph node metastases) in the narrow excision arm (hazard ratio (HR) = 1.26; P = 0.05) and a marginal difference between the two arms regarding DFS (HR = 1.21; P = 0.06). However, no difference in OS was observed (HR = 1.07; P = 0.6). Although these results are poorly understood and in contrast to all other trials, they briefly revived the discussion regarding 3-cm margins for melanomas thicker than 2 mm. This discussion now seems appeased by the report of the Scandinavian trial, which was presented at the 6th Melanoma World Conference in Vancouver, 6–10 September 2005. In this trial, 936 patients with melanomas thicker than 2 mm were randomised to either a 2-cm or 4-cm excision margin, and the results were virtually identical in all respects including locoregional and distant recurrences, and OS. 

### Regional lymph nodes

#### Failure of elective lymph node dissection to improve survival

Since the results of WHO trials 1 and 14 have been published, there has been minimal debate in Europe concerning radical lymph node dissection in melanoma patients, and SNB has eliminated any remaining ambiguity. Minor differences were described in SNB indications and methodology. Almost one-third of MUs agree to perform SNB at the request of the patient when the primary melanoma is thinner than 1 mm.

Although lymphatic and haematogenous spread usually occurs concurrently in most solid tumours including melanoma, and lymph node metastases are “indicators rather than governors of survival” [10], many patients with stage III disease will not develop distant metastases. On this basis, four randomised trials evaluated whether elective regional lymph node dissection (ELND) improves survival [11–14]. All four trials failed to demonstrate a survival benefit for ELND, and, as a result, this was largely abandoned in Europe. The WHO-14 trial suggested that patients with nodal micrometastases at ELND had an improved survival compared to those patients undergoing a TLND for clinically positive nodes [14]. This trial also supports SNB [15] as an ideal staging procedure. 

#### Sentinel node (SN) biopsy

SN staging is based on the well-supported hypothesis that melanoma lymphatic metastases follow an orderly progression through afferent lymphatic channels to the SN before spreading to other regional, non-sentinel nodes.

SN status is the most powerful prognostic indicator in melanoma patients. Studies demonstrate five-year OS rates between 89 and 93% for SN negative patients, and OS rates between 64 and 67% for SN positive patients [16–18].

Whether SN staging will improve survival remains to be seen. A recent study by Doubrovsky *et al*. [18] showed that SNB is superior to ELND due to different protocols in histopathological examination of lymph nodes. However, SNB did not improve survival compared to ELND in this matched control study. More importantly, the analysis of the MSLT-I trial does not confirm any survival benefit for SN staging in patients with high-risk primary melanomas. Survival rates at five years are 87 and 86% respective of whether or not a SNB was performed [19, 31]. Whether a completion lymph node dissection following a positive SNB improves survival is also unclear at present. MSLT II trial is designed to give an answer to this question. SN staging is useful for stratifying similar patients into randomised systemic adjuvant therapy trials to determine whether any treatment proposal may be of benefit [25]. SN staging may improve long-term locoregional control in the lymph node basin compared to patients who undergo TLND due to a reduced bulk of disease and an increased effect of immunotherapy [19]. Moreover, MSLT II trial evaluates the role of ultrasound of the regional lymph nodes, on SNB positive patients, by detecting small non-palpable lymph node metastases occurring during follow-up [26].

Whether the finding of a positive sentinel node must always be followed by a completion lymph node dissection (CLND) remains also an open question. Tumour load in the sentinel node plays here an important role. Recently, it was demonstrated that the presence of submicrometastasis (<0.1 mm, between 10 and 30 tumour cells) was never associated with the finding of additional positive nodes in the CLNDs [27]. This finding was corroborated by expanding this investigation to other major centres. Moreover, it was demonstrated that patients with these minimal tumour burdens identified in the SN had the same prognosis as SN-negative patients for locoregional, distant failure and death [27]. 

#### No evidence SN-staging induces locoregional recurrence rates

Various studies have reported an increased incidence of in-transit metastases following SNB [20]. However, these studies included major discrepancies in primary tumour characteristics such as Breslow thickness and ulceration and improved studies demonstrate that the apparent increase is due to a prolonged recurrence free interval. Since SNB reduces nodal recurrences, it is more likely that the initial metastases will be in-transit and the overall probability unchanged regardless of whether early or delayed lymphadenectomy is performed [21–24]. The lymphadenectomy becomes of paramount concern once nodal metastases are diagnosed, as this procedure may be the final chance of cure for many patients with melanoma. Whether an aggressive dissection reduces locoregional or distant recurrences is difficult to postulate, but certainly if the regional nodes are dissected naive, the risk of local recurrence could be higher and the prognosis worse. An anatomical lymph node dissection should include all the nodes of that particular basin for histopathological examination. The axillary dissection includes all three levels of Berg nodes. For unknown reasons, many centres limit the dissection to levels I and II even though sparing the remaining level III nodes will not further reduce the low incidence of lymphoedema of the upper limb. In the neck, the nodes involved include levels I to V with or without a superficial parotidectomy. Twelve centres perform a limited dissection for nodal micrometastases confirmed by IHC only (Table 3). There was no general consensus regarding the indication to perform a superficial parotidectomy for primary melanoma located between the zygoma and the mastoid and some centres do not perform a parotidectomy if there is no evidence of infraparotid metastases.

Groin dissection is the most typical surgical procedure for a melanoma surgeon. There is ongoing controversy regarding whether to extend the dissection to include the pelvic nodes or limit it only to the superficial and deep inguinal nodes. At least two-thirds of MUs do not proceed with a pelvic node dissection. One-third of MUs analyse Cloquet’s node, but evidence suggests that the status of this node does not mirror metastases to the pelvis.[33] One-third of MUs do not dissect grossly normal pelvic nodes because of the increased morbidity associated with a pelvic node dissection including lymphoedema of the lower limbs and a more complicated post operative recovery. 

#### In-transit metastases and surgery for stage IV disease

ILP was performed by one-third of MUs. Of concern is the fact that three centres continue to perform this procedure as an adjuvant to surgery after a randomised trial previously demonstrated that this did not improve prognosis [28]. Prophylactic ILP following surgery for patients with primary melanoma at high risk of recurrence was popular in Europe because retrospective studies had suggested improved outcomes. The intergroup trial (EORTC 18832/WHO-15) randomised 832 patients and demonstrated that prophylactic ILP had a regional effect reducing the incidence of in-transit metastases from 6.6 to 3.3% and regional lymph node metastases from 16.7 to 12.6%, but had no effect on distant relapse-free or OS [28]. Accordingly, prophylactic ILP should no longer be performed or reimbursed in Europe: there is no justification to explain the indication offered by three MU for this approach. However, ILP with melphalan and TNF is highly effective for multiple or bulky symptomatic in-transit metastases, with CR rates of 70% and similar response rates following treatment failure with melphalan alone [29, 30, 31]. Isolated limb infusion was not investigated in this survey.

Surgery for stage IV disease was proposed by 50% of centres, with the principal objectives being the resection of all documented disease and palliation of symptoms.

Even if the participation to our survey was lower (45%) than expected, the MUs surveyed were the most important in terms of clinical activity. 

## Conclusions

The important surgical oncological question over the past 50 years have been to move from a concept of the most extensive demolition to the most conservative surgical approach that could guarantee the same oncological result but a higher result in quality of life.

Adequacy of surgical resection margins for primary melanoma and the best approach regarding regional nodes are the two most important issues concerning many randomised trials.

Studies to determine adequate surgical margins for primary melanoma did not show any survival difference on the basis of the width of resection. The four historical and most important studies conducted, always showed that the results following a narrow excision margin were equal to a wider margin [1, 3, 4, 8]. The UK MG demonstrated a reduced risk of in-transit metastases after wide excision, but no impact on OS. A similar approach was followed in several trials investigating the importance of performing an immediate dissection of regional nodes in the clinical absence of metastases [5, 16]. Since SNB has become standard of care by most centres, a revolution has invaded the treatment guidelines regarding locoregional nodes. The wait and see policy of the trials already mentioned has been overtaken *de facto *by a more specific and selective invasive approach to obtain precise staging of clinically negative regional nodes. SNB has concentrated most of the clinical research tools required in surgery including conservation, precision, and sophistication, and is the best indication whether a radical TLND is required. Analysis of data from this questionnaire shows a sensible and acceptable difference in surgical practice between the various MUs.

The evidence to date suggests that surgical margins for primary melanoma should be 1 or 2 cm [1–18]. The indications and degree of lymph node dissection also varied between MUs, and currently there is no evidence to suggest whether a less invasive dissection impacts survival [19].

Similarly, the indication for surgery in patients with stage IV disease varied among the MUs, and there is little evidence to define the role of surgery in this patient group. The EORTC-WHO 15 study, however, demonstrated that ILP is ineffective as an adjuvant to surgery for primary melanomas thicker than 1.5 mm and should not be performed on such indication [28].

The adequacy of surgery appears to be the most important milestone in the therapeutic history of melanoma. Surgery is fundamental in the initial stages of the disease including definitive treatment of primary lesions and locoregional disease. However, there is limited information regarding appropriate surgical treatment in the presence of lymph node metastases and if a radical lymph node dissection improves prognosis [24].

Phase III randomised trials have shown that wide margins, elective lymph node dissections and prophylactic isolated limb perfusions have not improved survival and cannot be considered standard of care in the routine management of primary melanoma. Finally, SNB is an excellent diagnostic procedure and play an important prognostic role in melanoma patients [32]. 

Although this analyses was performed between 2003 and 2005, it could be useful to analyse the differences in the approach of melanoma patients in each surgical scenario of the disease. The surgical subgroup of the EORTC MG is developing a new version of the surgical survey questionnaire including new treatment modalities like isolated limb infusion and electrochemotherapy which were not frequently in use some years ago. Once completed, an interesting aspect will be to compare the differences developed during a ten-year period concerning the surgical management of melanoma patients. 

## Figures and Tables

**Table 1.** Percentages of units (n = 75) indicating melanoma resections margins by AJCC stage

		**AJCC**				
	**Margins (cm)**	***In Situ***	T1	T2	T3	T4
Precise answers	0.2	5				
	0.2–0.5	4				
	0.5	71	5			
	0.5–1	4	3			
	1	12	88	30	3	3
	1.5			4		
	1–2			10	3	3
	2		1	53	71	49
	2–3			1	8	5
	2–4				3	3
	3			1	9	28
	3–4				1	1
	4				1	4
	Not filled	4	3	1	1	4
	Total	100	100	100	100	100
Intervals (including extremes)	0.2–0.5	80				
	0.5–1	96	96			
	1–2		89	97	77	55
	2–3			55	88	82
	2–4			54	85	90

**Table 2.** Frequencies (%) of SNB for Breslow thickness <1 mm, according to the EORTC membership status

SNB	Active (AM)	Probationary (PM)	Foreign (FMU)	All
Patient choice	6 (19)	14 (44)	12 (38)	32 (100)
Regression	11 (24)	23 (50)	12 (26)	46 (100)
Ulceration	12 (24)	25 (50)	13 (26)	50 (100)
Clark IV–V	11 (26)	19 (45)	12 (29)	42 (100)
Nodular melanoma	7 (37)	8 (42)	4 (21)	19 (100)
All	21 (28)	35 (47)	19 (25)	75 (100)

**Table 3.** Type of axillary dissection depending on the SNB pathology report: SNB REPORT

Lymphatic basin	Lymphadenectomy	Macro-metastases	Micro-metastases	IHC+	PCR+
Neck	Modified rad.	52	51	35	2
	Radical	8	2	1	0
	Limited	3	10	12	2
	Not performed	0	0	15	59
	Not filled	0	0	0	0
Axillary	I	0	1	3	0
	I + II	11	19	15	3
	I + II + III	50	39	30	2
	No dissection			9	38
	Not filled	2	4	6	20
Groin and iliac	IIO	41	38	27	3
	IID. Cloq+	21	16	13	1
	SID	1	7	9	2
	No		2	11	45
	Not Filled	0	0	3	12
Total		63	63	63	63

**Table 4:** Surgical margins (trials).

Trial, year	No. of subjects	Resection margins	Breslow thickness, mm	Overall survival narrow, wide	Local recurrence rate narrow, wide
French. Multicentric, Trial 1993	**319**	**2-5**	**< 2**	**87%, 86%**	**13.6%, 20%**
Intergroup Melanoma Trial, 1996	**470**	**2–4**	**1–4**	**70%, 77%**	**2.1%, 2.6%**
Swedish MSG Trial, 2000	**989**	**2–5**	**0, 8–2**	**79%, 76%**	**0.6%, 2.4%**
WHO Melanoma Trial 10, 1991	**612**	**1–3**	**< 2**	**96.4%, 96.4%**	**0.98%, 0.97%**
UK Melanoma Study Group, 2004	**900**	**1–3**	**> 2**	**32.2%, 30.9%**	**8.27%, 5.64%**

## Appendix

### SURGICAL GUIDE LINES

**A. - MELANOMA RESECTION MARGINS**

1. Please fill the spaces: **Margins in cm**

**Table:**

	American Joint Committee on Cancer 2002					
**Primary mm**	**In situ**	**T1 < 1mm**	**T2 1–2 mm**	**T3 2–4 mm**	**T4 >4 mm**	**Satellites**
Margins						

**Margins:** Fill with the size of the margins according to the size of the tumor (AJJCC 2002)

**B. - SENTINEL NODE BIOPSY.**

**Do you perform SNB? Do you use Patent Blue and Gamma Probe?: Yes or No**

**Table:**

SNB	PATENT BLUE	GAMMA PROBE
Yes □ No □	Yes □ No □	Yes □ No □

**SNB:** If you perform Sentinel Node Biopsy, at which tumor thickness do you perform it?

**Table:**

PRIMARY	SNB
Thickness < 1 mm	Yes □ No □
Thickness < 1-4 mm	Yes □ No □
Thickness > 4 mm	Yes □ No □
**If presence of primary melanoma < 1mm. Do you perform as well SNB? if:**
Nodular Melanoma	Yes □ No □
Clark IV-V	Yes □ No □
Ulcerated	Yes □ No □
Regression	Yes □ No □
Patient choice	Yes □ No □

If you want to add some information, please do it below:

**Table:**

**C.- AXILLARY, NECK AND ILIO-INGUINAL DISSECTION**

***LIMPHNODE METASTASIS LEGEND:*** **Micrometastasis:** < 2 mm **IHC:** Immunohistochemistry **PCR:** Polymerase Chain Reaction

***NECK DISSECTION LEGEND:*** **Modified Radical Neck Dissection:** Levels I - V; Conservative of sternocleidomastoid muscle, spinal accessory nerve and internal jugular vein. **Radical Neck Dissection:** Levels I - V; Demolitive of sternocleidomastoid muscle, spinal accessory nerve and internal jugular vein. **Limited ( Selective) Neck Dissection:** Not all I - V levels are dissected. **Parotidectomy + Neck Dissection:** ***Case 1:*** In presence of cyto-histologically documented infraparotid metastasis. ***Case 2:*** If primary tumor is between zigomatic and mastoid.

1. Please fill the spaces describing your surgical approach in the four different situations: **Yes or No**

**Table:**

AXILLARY DISSECTION	Macro MT+	Micro MT+	Only IHC+	Only PCR+
Level I	Yes □ No □	Yes □ No □	Yes □ No □	Yes □ No □
Levels I + II	Yes □ No □	Yes □ No □	Yes □ No □	Yes □ No □
Levels I + II + III	Yes □ No □	Yes □ No □	Yes □ No □	Yes □ No □
**ILIOINGUINAL DISSECTION**				
Superficial Inguinal	Yes □ No □	Yes □ No □	Yes □ No □	Yes □ No □
Deep Inguinal	Yes □ No □	Yes □ No □	Yes □ No □	Yes □ No □
Ilio Obturatory	Yes □ No □	Yes □ No □	Yes □ No □	Yes □ No □
Ilio Obturatory only if Cloquet +	Yes □ No □	Yes □ No □	Yes □ No □	Yes □ No □
**NECK DISSECTION**				
Modified Radical	Yes □ No □	Yes □ No □	Yes □ No □	Yes □ No □
Radical	Yes □ No □	Yes □ No □	Yes □ No □	Yes □ No □
Limited	Yes □ No □	Yes □ No □	Yes □ No □	Yes □ No □

**Table:**

Parotidectomy + Neck Dissection in case 1 Yes □ No □
Parotidectomy + Neck Dissection in case 2 Yes □ No □

**D.- LIMB PERFUSION.**

**Do you perform limb perfusion?** □Yes   No □

If **yes** go to **D1**

If **No** go to **D2**

**D1.** *For Centers doing Limb Perfusion:*

***ADJUVANT LIMB PERFUSION (ALP) LEGEND:*** ( As proposed by one of the group members ) 1. Primary MM of specific worsen prognosis (sole, subungueal), in particular if associated with SNB +. 2. High risk primary MM with satellitosis on ulceration or inadequate surgical excision. 3. High risk primary MM with uncertain possibility of follow - up. 4. High risk primary MM without alternative therapeutic options.

1. Please fill the spaces: **Yes or No**

**Table:**

	Limb Perfusion	ALP
**Adjuvant**	Yes □ No □	1 □ 2 □ 3 □ 4 □
**Therapeutic**	Yes □ No □
**At first in transit MTs**	Yes □ No □
**Only if in transit MT are not operable or rapidly recurring**	Yes □ No □

If you want to add some information, please do it below:

**Table:**

**D2.***For Centers not doing Limb Perfusion:*

**Do you refer patients to a center doing Limb Perfusion?** □Yes   No □

□ At first in transit Metastasis.

**If yes:** When do you refer a patient for Limb Perfusion?

□ Only if in transit MT are not operable or rapidly recurring.

□ Surgery

**If No:** What do you indicate?

□ Systemic therapy.

If you want to add some information, please do it below:

**Table:**

**E.- SURGERY ON DISTANT METASTASIS.**

1. Please fill the spaces: **Yes or No**

**Table:**

Only if Superficial	Yes □ No □
Only Single Visceral MT	Yes □ No □
Multiple Visceral MT	Yes □ No □
Only for emergency	Yes □ No □
Only if required in clinicals trials	Yes □ No □
Also for symptomatic palliation	Yes □ No □

If you want to add some information, please do it below:

**Table:**
